# Acute pulmonary embolism in South Africa

**DOI:** 10.7196/SARJ.2018.v24i3.224

**Published:** 2018-09-07

**Authors:** Richard Raine

**Affiliations:** Respiratory Clinic, Groote Schuur Hospital and University of Cape Town, South Africa

## Editorial

Pulmonary embolism is a common problem associated with significant morbidity and mortality. In this issue of the *AJTCCM*, Meel *et al*. ^[1]^ report on a retrospective analysis of patients with pulmonary embolism diagnosed by computed tomographic pulmonary arteriography (CTPA) at an academic hospital in Gauteng Province.

The diagnosis was made in 30% (n=147/498) of CTPA procedures. This diagnostic rate compares favourably with European figures (20% - 30%), and is far better than the rate of <10% reported in the USA.^[2]^ Concerns have been raised about the overuse of CTPA, which is not necessarily a benign procedure. There are potential risks associated with administration of contrast, including kidney injury and anaphylaxis, as well as radiation exposure to breast and other tissues. Overdiagnosis and overtreatment have been described, with complications related to unnecessary anticoagulation in individuals with isolated subsegmental pulmonary emboli.^[3]^ The diagnostic rate reported by Meel *et al*. ^[1]^ is acceptable, particularly when CTPA often gives other information on these patients, many of whom are HIVreactive and have other infections or disease processes.

Recommendations from a number of authorities include the use of a pretest probability score (Wells Score or Revised Geneva Score) and a highly sensitive D-dimer test prior to performing CTPA. These, however, are often overlooked, with <50% of CTPA requests following guideline recommendations.^[4]^ Meel *et al*. ^[1]^ show similar results, with Wells scores and D-dimer test results reported for ~60% (n=88/147) and ~52% (n=77/147) of CTPA-proven pulmonary emboli, respectively. No data were available for the cases not showing pulmonary emboli, so it was not possible to assess the utility of CTPA procedures in this cohort, as there were no data for procedures in which no pulmonary emboli were found. The comorbidities reported included HIV-reactivity and tuberculosis, which are unlikely to feature highly in European or North American studies.

Mortality was high, with 24% of patients dying, although fewer than half had features of being at high risk for mortality from pulmonary embolism. This suggests that pulmonary embolism was likely to be discovered in patients who were already severely ill. Thrombolytic therapy was administered to only 15% of patients who met the criteria for being at high risk of mortality. Thrombolysis was withheld in many patients owing to the presence of comorbidities and uncertainty about haemodynamic status. Although thrombolysis is undoubtedly the treatment of choice for patients with pulmonary embolism who are at high risk for mortality, the role of thrombolysis in moderate-risk patients remains controversial.^[5]^ Those dealing with acute pulmonary embolism should be aware of the benefits of thrombolysis in the highrisk group, and be prepared to administer this potentially life-saving therapy. The findings of the study of a local population by Meel *et al*. ^[1]^ would be very useful in assessing the applicability of international guidelines to local practice.
